# Sickness-certification practice in different clinical settings; a survey of all physicians in a country

**DOI:** 10.1186/1471-2458-10-752

**Published:** 2010-12-06

**Authors:** Christina Lindholm, Britt Arrelöv, Gunnar Nilsson, Anna Löfgren, Elin Hinas, Ylva Skånér, Anna Ekmer, Kristina Alexanderson

**Affiliations:** 1 Department of Clinical Neuroscience, Division of Insurance Medicine, Karolinska Institutet, SE-171 77 Stockholm, Sweden; 2 Stockholm County Council, SE-118 91 Stockholm, Sweden; 3 Department of Neurobiology, Care Sciences and Society, Centre for Family and Community Medicine, Karolinska Institutet, 141 83 Huddinge, Sweden

## Abstract

**Background:**

How physicians handle sickness-certification is essential in the sickness-absence process. Few studies have focused this task of physicians' daily work. Most previous studies have only included general practitioners. However, a previous study indicated that this is a common task also among other physicians. The aim of this study was to gain detailed knowledge about physicians' work with sickness-certification and of the problems they experience in this work.

**Methods:**

A comprehensive questionnaire regarding sickness-certification practice was sent home to all physicians living and working in Sweden (N = 36,898; response rate: 61%). This study included physicians aged <65 years who had sickness-certification consultations at least a few times a year (n = 14,210). Descriptive statistics were calculated and odds ratios (OR) with 95 % confidence intervals (CI) were estimated for having different types of related problems, stratified on clinical settings, using physicians working in internal medicine as reference group.

**Results:**

Sickness-certification consultations were frequent; 67% of all physicians had such, and of those, 83% had that at least once a week. The proportion who had such consultations >5 times a week varied between clinical settings; from 3% in dermatology to 79% in orthopaedics; and was 43% in primary health care. The OR for finding sickness-certification tasks problematic was highest among the physicians working in primary health care (OR 3.3; CI 2.9-3.7) and rheumatology clinics (OR 2.6; CI 1.9-3.5). About 60% found it problematic to assess patients' work capacity and to provide a prognosis regarding the duration of work incapacity.

**Conclusions:**

So far, most interventions regarding physicians' sickness-certification practices have been targeted towards primary health care and general practitioners. Our results indicate that the ORs for finding these tasks problematic were highest in primary health care. Nevertheless, physicians in some other clinical settings more often have such consultations and many of them also find these tasks problematic, e.g. in rheumatology, neurology, psychiatry, and orthopaedic clinics. Thus, the results indicate that much can be gained through focusing on physicians in other types of clinics as well, when planning interventions to improve sickness-certification practice.

## Background

Physicians have an essential role in the sickness absence process; however, there is very little scientific knowledge about this. In the last years, some more studies have been published but the number still is scarce [1-6]. Possible negative consequences of being sickness absent have recently been highlighted [7,8] as well as the importance of sickness certificates being issued with the same caution as other recommendations made by physicians to their patients, in order to avoid negative consequences [1,9,10].

In most countries there are two requisites for being entitled to sickness benefits; one must have a disease or an injury and this disease or injury must have affected one's work capacity [11]. In Sweden, as in most countries, all physicians may issue sickness certificates. However, writing the certificate is only one of the tasks that may be involved in a consultation where sickness-certification is considered. Those different tasks can be summarised as [1]:

• Determine if the patient has a disease or an injury.

• To ascertain whether the disease or injury impairs the patient's functional ability to the extent that the work capacity is also impaired in relation to her or his work demands.

• Consider, together with the patient, the possible advantages and disadvantages of being sickness absent.

• Determine the duration and grade (full or part time) of sick leave and the medical investigations, treatments, or other measures needed during the sick-leave period.

• Determine possible needs for contact with other specialists, the social insurance office, occupational health services, the employer, or other stakeholders and if so, to establish adequate communication.

• Issue a certificate that provides sufficient information for the employer or social insurance officer to decide whether the patient is entitled to sickness benefits and in need of further return-to-work measures.

• Document assessments and actions taken.

In a systematic literature review of studies of sickness-certification practice, scientific evidence could be established for only two issues; that the sickness certificates had low quality and that the physicians found sickness-certification problematic [1,11]. The Swedish Council on Technology Assessment in Health Care (SBU) concluded that the number of studies was low and that sample sizes were small and often very biased. Moreover, SBU stated that larger studies were warranted to be able to generalize results and to get more detailed information, as basis for interventions.

In this research area it is especially crucial to also use the physicians' perspective on the sickness-certification task and related problems, in order to be able to tailor interventions along the line of their experiences and wishes. Moreover, most studies in this area have included only general practitioners (GPs) [2,3,11-27]. Some studies have compared the frequency and the severity of problems among GPs with that of other physicians [1,5,6,28-30]. Nevertheless, the knowledge about sickness-certification practice among other physicians than GPs is still scarce.

Therefore, we in a previous questionnaire study included physicians also from other clinical settings [6]. One finding was that consultations involving sickness-certification were equally, or even more, frequent among other specialists and clinics than in primary health care (PHC)/among GPs [6,29]. This was unexpected and we have, consequently, here pursued our research about this in a larger study, including all the physicians in Sweden.

The aim was to gain detailed knowledge about physicians' work with sickness-certification regarding frequency of different types of situations and severity of related problems, in general as well as in specific clinical settings.

## Methods

A cross-sectional questionnaire study was conducted. The questionnaire was administered to all physicians in Sweden, a country with 9.3 million inhabitants. The study population was the 36,898 physicians of all ages who lived and mainly worked in Sweden in October 2008. They were identified using a register of all physicians, held by the company that in Sweden has the most complete such register; Cegedim AB.

A comprehensive questionnaire of 163 questions about various aspects of sickness-certification practice and related work issues was developed, based on a previous questionnaire, research, and contacts with many different clinicians and researchers in the area [29,31]. The questionnaire was tested in a pilot study with a random sample of 100 physicians (62.0% response rate).

The questionnaire was distributed by mail to the participant's home addresses in order to avoid interaction with colleagues in completing the questionnaire. Three reminders were sent to non-responders. Distribution, registration, scanning of questionnaires, and basic management of data was administered by Statistics Sweden. The response rate was 60.6%. There was no large bias in the drop-out; as expected the response rate was somewhat higher among women and older physicians (Table 1).

**Table 1:** Study population characteristics, response rate, number and proportion in clinical settings having sickness-certification consultations.

	Study population (N)	Responders (n, %)		Responders having sickness-certification consultations at least a few times a year (n, %)	
All	36898	22349	60.6	15057	67.4
					
Women	15946	10085	63.2	6946	68.9
Men	20936	12259	58.6	8110	66.2
Missing information	16	5	31.3	1	20.0
					
24-44 year	14462	8349	57.7	6172	73.9
45-64 year	19898	12110	60.9	8038	66.4
65+ year	2538	1890	74.5	847	44.8
					
Specialist	26242	16300	62.1	10607	65.1
Non-specialist	10656	6049	56.8	4450	73.6
					
*Clinical settings1*					
Primary health care (PHC)		4394		4278	97.4
Internal medicine		2121		1963	92.5
Child and adolescent care		1665		409	24.6
Surgery		1562		1376	88.1
Psychiatry		1284		1142	88.9
Gynaecology		1070		930	86.9
Administration		1055		103	9.8
Orthopaedic		939		898	95.6
Occupational health service		500		482	96.4
Ophthalmology		489		358	73.2
Ear, nose, and throat		486		445	91.6
Geriatrics		458		121	26.4
Infectious diseases		342		331	97.0
Oncology		348		334	96.0
Dermatology		265		208	78.5
Neurology		259		244	94.2
Rheumatology		193		191	99.0
Rehabilitation		190		177	93.2
Pain management		112		85	75.9
Other		3184		880	27.6
Do not work in a clinic		709		46	6.5
Missing		724		56	7.7

*^1^*Information about response rate in different clinics is not available.

In this study we included the 14,210 participants who were below the age of 65 and had consultations concerning sickness-certification at least some times in a year.

### Data sources

Information about age, sex, and being a board certified specialist was provided by the National Board of Health and Welfare via Cegedim AB. All other data were provided by the questionnaire. Information about type of clinical setting the participant mainly worked in is presented for 18 specified clinics, "other clinics", and "administration/research/education" (in tables and figures called "Administration"), respectively.

Frequency of consultations, frequency of related problems, and severity of experienced problems, respectively, were measured by answers to three questionnaire items:

*- Frequency of consultations *concerning sickness-certification was measured by the alternative answers; "more than 20 times a week", "6-20 times a week", "1-5 times a week", "about once a month", "a few times a year", and "never or almost never". The two response alternatives "about once a month" and "a few times a year" were combined to "less than once a week".

- The response alternatives regarding *frequency of problems *in handling sickness-certification consultations were; "more than 10 times a week", "6-10 times a week", "1-5 times a week", "about once a month", "a few times a year", and "never or almost never". The alternatives "about once a month" and "a few times a year" were combined to "less than once a week".

- Regarding *severity of experienced problems*, the response alternatives were "very", "fairly", "somewhat", and "not at all" to a generic question "How problematic do you generally find it to handle sickness-certification of patients?" and to 16 specified problems.

### Statistics

Results from descriptive statistics of frequencies of consultations and problems regarding those, the association between them, as well as the severity of problematic tasks were stratified by type of clinic. Analysis made of the variations between age groups and between men and women turned out to be small and the results appear, therefore, for all together.

The odds ratios (OR) of having very or fairly problematic situations were estimated with 95% confidence interval (CI), adjusted for specialist/not specialist. The reference group was physicians working in internal medicine clinics, chosen because of its large size and moderate level of problems. ORs were calculated for items that at least half of the responders found very or fairly problematic. The analyses were performed using the SPSS 17.0 program.

The study was approved by the Regional Ethical Review Board of Stockholm.

## Results

Of all the responding physicians below the age of 65, 67.4% had consultations considering sickness-certification at least a few times a year. That rate was higher for non-specialists than for specialists; 73.6% and 65.1%, respectively (Table 1). In a number of clinical settings, nearly all physicians had such consultations at least a few times a year; PHC (97.4%), infectious diseases (97.0%), occupational health service (96.4%), oncology (96.0%), and rheumatology (99.0%).

**Figure 1:**
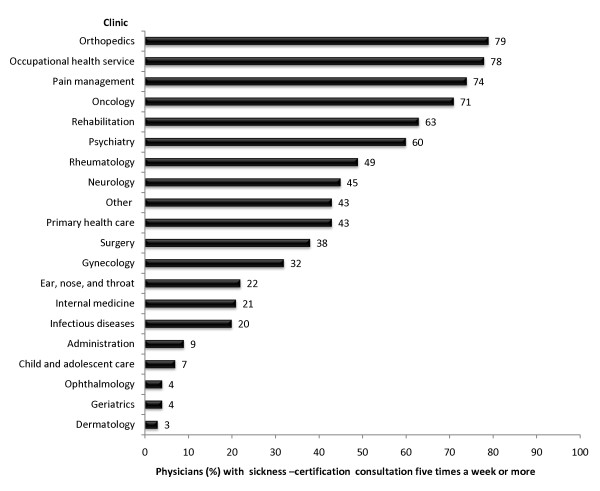
**Physicians (%) in clinical settings having sickness-certification consultations more than five times a week**.

From here on, only those 14,210 physicians <65 years of age who at least sometimes per year had sickness-certification consultations were included in the analyses. More than one third of the physicians in twelve types of clinics had such consultations more than five times a week (Figure 1). The highest proportion of physicians who had such consultations more than five times a week were physicians in orthopaedic clinics and occupational health service. Notably is that also pain management, oncology, psychiatry, rehabilitation, rheumatology, and neurology, together eight different clinics, had much higher rates of sickness-certification consultations than those in PHC.

Frequency of sickness-certification consultations as well as of related problems varied substantially with type of clinic. Characteristics of the clinics regarding mean age, sex, and proportion of board certified specialists varied as well (Table 2).

**Table 2:** Physicians' characteristics, frequencies of sickness-certification consultations and related problems (%), distributed on clinical settings.

	Characteristics of the physicians^a ^ in different clinics			Rate (%) of physicians^a ^who had sickness-certification consultations at different frequencies during a week				Rate (%) of physicians^a ^who experienced problems related to sickness-certification at different frequencies during a week			
Clinical setting	Mean age	Women %	Specialist %	> 20 times a week	60-20 times a week	1-5 times a week	< once a week	> 10 times a week	6-10 times a week	1-5 times a week	< once a Week
Internal medicine	43	45.4	58.9	2.4	19.1	50.0	28.5	0.2	1.6	14.8	83.4
Primary health care	48	51.0	66.8	2.3	40.4	52.4	4.9	2.2	5.5	46.6	45.8
Surgery	44	30.7	64.7	4.6	33.4	48.1	13.8	0.8	1.5	15.9	81.9
Psychiatry	47	55.5	65.6	10.0	50.0	30.6	9.4	3.5	7.2	39.3	50.0
Gynaecology	47	69.0	74.1	1.8	29.6	50.6	18.0	0.8	3.5	22.0	73.7
Orthopaedic	45	20.8	67.9	19.2	59.3	18.5	3.1	3.5	7.5	42.5	46.5
Occupational health	56	43.8	95.1	16.3	62.0	18.9	2.8	2.6	6.7	35.1	55.6
Ear, nose, and throat	47	44.0	77.1	0.9	21.0	60.0	18.0	0.2	1.0	11.4	87.4
Child and adolescent	48	53.9	78.4	0.8	5.7	22.4	71.1	0.3	0.3	8.1	91.3
Ophthalmology	48	56.8	80.3	0.0	3.8	21.8	74.4	0.0	0.3	4.0	95.7
Oncology	45	57.8	64.9	20.2	50.9	23.9	5.0	0.6	1.6	16.4	81.4
Infectious diseases	43	49.7	62.9	1.6	18.6	60.7	19.2	0.3	1.0	14.1	84.7
Neurology	46	42.2	75.4	2.6	42.7	47.8	6.9	0.0	3.1	37.0	59.9
Dermatology	48	68.0	77.0	0.0	3.5	22.0	74.5	0.5	0.5	7.4	91.5
Rheumatology	49	53.8	79.1	3.3	45.6	45.6	5.5	1.7	4.4	39.2	54.7
Rehabilitation	51	57.7	79.2	17.9	45.2	26.2	10.7	1.9	2.5	25.3	70.4
Geriatrics	49	57.0	78.9	0.9	3.5	14.0	81.6	0.0	0.0	1.9	98.1
Administration	53	42.7	86.6	4.9	3.7	31.7	59.8	1.5	0.0	7.7	90.8
Pain management	54	28.8	90.4	17.8	56.2	20.5	5.5	6.3	12.7	23.8	57.1
Other	48	43.4	72.7	15.7	26.7	32.8	24.8	1.9	2.5	15.0	80.6
Do not work in a clinic	41	50.0	47.4	13.2	36.8	28.9	21.1	3.6	7.1	32.1	57.1
*All of the physicians*^a^	*47*	*47.6*	*68.8*	*5.8*	*34.5*	*42.5*	*17.2*	*1.6*	*3.8*	*28.9*	*65.7*
											
Missing (n = 56)	43	44.6	51.8	10.7	21.4	48.2	19.6	2.2	2.2	22.2	73.3

^a ^Physicians (aged <65) who had sickness-certification consultations at least a few times a year, n = 14,210

**Figure 2:**
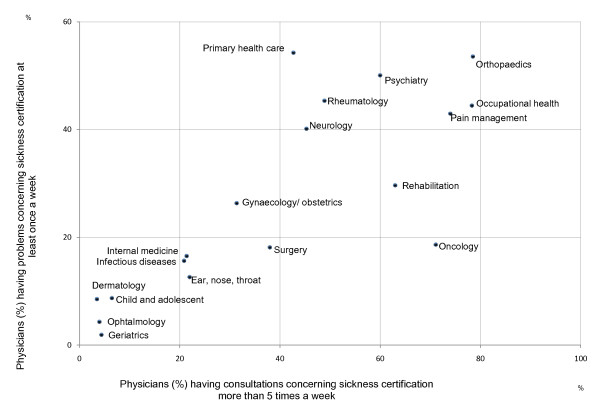
**Sickness-certification consultations and problems regarding this among physicians (%) in clinical settings**.

The association between the proportion of physicians in different clinics having sickness-certification consultations more than five times a week and the proportion having problems with these work tasks at least once a week is illustrated in Figure 2.

In most clinics, the proportion of physicians having many such consultations was positively associated to the proportion finding them problematic. However, the physicians in oncology clinics and in PHC diverged. A high rate of the physicians in oncology clinics had sickness-certification consultations but a relatively low rate found them problematic, while the contrary was found for PHC.

The physicians were asked to assess how problematic they experienced 16 different situations (Table 3). The task most physicians (59.9%) rated as fairly or very problematic was to assess the patient's work capacity followed by providing a long-term prognosis for the duration of the work incapacity (58.4%). Moreover, a high percentage found it problematic to handle prolongations of a sick-leave period that initially had been certified by a colleague (52.3%) and to provide social insurance officers with other types of medical certificates, e.g. for disability pension (49.9%). The least problematic task was to know what to document in the medical chart, 43.2% stated that this was not at all problematic.

**Table 3:** The physician's assessment of how problematic they experienced different sickness-certification tasks to be (%).

How problematic do you generally find it to...	*Very* %	*Fairly* %	*Somewhat* %	*Not at* *all *%
...handle sickness-certification of patients?	6.8	33.1	43.2	17.0
...assess whether a patient's functional capacity is reduced?	14.6	33.7	37.3	14.4
...assess whether the reduced functional capacity is due to disease or injury?	10.0	28.3	41.5	20.1
...assess the degree to which the reduced functional capacity limits a patient's work capacity?	22.6	37.3	30.8	9.3
...provide a long-term prognosis about the future work capacity of sick-listed patients?	22.3	36.1	28.2	13.4
...assess the optimum duration and degree of sickness absence?	16.2	36.7	36.1	11.0
...suggest a plan of action and/or measures to be taken during the sick leave?	8.9	26.0	37.4	27.7
...decide whether to certify a prolongation of a sick- leave period initially certified by another physician?	17.8	34.5	32.7	15.0
				
...manage your dual role of being a doctor for your patients and a medical expert for the Social Insurance Office and other authorities?	16.3	28.4	32.5	22.9
...discuss possible changes in lifestyle and life circumstances with a patient who is being issued a sickness certificate?	8.0	29.4	38.8	23.8
...discuss and know how to deal with other psychosocial problems (e.g. economic difficulties or physical or substance abuse) when managing a patient on sick leave?	12.0	28.5	36.1	23.4
...manage situations in which you and a patient have different opinions about the need for sickness absence?	15.6	30.7	37.4	16.3
...discuss with the patient the advantages and disadvantages of being on sick leave?	4.7	23.3	44.9	27.0
				
...issue sickness certificates for the social insurance officers?	10.8	26.4	38.5	24.4
...provide the SIO with other types of certificates?	16.6	33.3	31.9	18.2
...know what aspects of the sickness-certification process that is to be documented in the patient's chart?	4.3	14.9	37.6	43.2
...handle situations in which you and other members of the healthcare team have different opinions about sickness-certification of a patient? (Not applicable for 44.7%)	2.7	9.2	23.9	19.6

The OR of having problems in handling sickness-certification cases was, using physicians in internal medicine as reference group, highest among physicians in PHC; OR 3.3, 95% CI 2.9 to 3.7 and in rheumatology clinics; OR 2.6, 95% CI 1.9 to 3.5 (Table 4). Physicians in neurology, pain management, orthopaedics, and psychiatric clinics also had an OR above 1.0. Physicians in PHC had significantly higher OR for all the specified problems while physicians in pain management didn't have OR above 1.0 for any of the specified problems. Physicians in psychiatric, neurologic, or orthopaedic clinics had higher ORs for experiencing it very problematic to assess and to prognosticate patients' work capacity. Physicians working in orthopaedic clinics had a slightly higher OR for finding it problematic to prolong a sick-leave period initially certified by another physician.

**Table 4:** Physicians (%) who experienced sickness-certification as very or fairly problematic stratified on clinics.

**How problematic do you generally find it to..**.										
	...handle sickness- certification of patients?		...to assess the degree which the reduced functional capacity limits a patient's work capacity?		...provide a long-term prognosis about the future work capacity of a sick-listed patient?		...assess the optimum duration and degree of sickness absence?		...decide whether certify a prolongation of a sick-leave period initially certified by another physician?	
Clinical setting:	%	OR^b ^(95% CI)	%	OR^b ^(95% CI)	%	OR^b ^(95% CI)	%	OR^b ^(95% CI)	%	OR^b ^(95% CI)
Internal medicine^a^	33.2	1	57.9	1	58.2	1	54.1	1	53.0	1
Primary health care	60.5	**3.30 (**2.93-3.72)	81.5	**3.51 **(3.09-3.98)	78.2	**2.80 **(2.48-3.17)	70.0	**2.15 **(1.91-2.42)	70.0	**2.17 **(1.93-2.43)
Rheumatology	50.5	**2.57 **(1.87-3.52)	64.3	**1.61 **(1.16-2.22)	66.3	**1.65 **(1.19-2.29)	52.8	1.20 (0.88-1.65)	56.9	1.33 (0.97-1.81)
Psychiatry	47.2	**1.96 **(1.67-2.31)	61.9	**1.28 **(1.08-1.50)	70.5	**1.83 **(1.54-2.16)	55.7	1.16 (0.98-1.36)	47.5	**0.83 **(0.71-0.97)
Neurology	44.0	**1.88 **(1.41-2.51)	61.1	**1.35 **(1.01-1.81)	63.3	**1.42 **(1.06-1.90)	55.1	1.29 (0.96-1.72)	49.6	0.97 (0.73-1.28)
Pain management	38.1	1.66 (0.98-2.82)	58.1	1.35 (0.80-2.27)	63.5	1.56 (0.92-2.64)	45.2	0.99 (0.59-1.66)	44.4	0.86 (0.51-1.43)
Orthopaedic	42.7	**1.61 **(1.36-1.92)	61.0	**1.24 **(1.04-1.47)	61.0	**1.20 **(1.02-1.43)	52.6	1.04 (0.87-1.23)	56.5	**1.23 **(1.04-1.45)
Occupational health	28.6	1.14 (0.89-1.45)	41.6	**0.72 **(0.58-0.90)	43.1	**0.70 **(0.56-0.88)	36.0	**0.71 **(0.57-0.90)	38.9	**0.70 **(0.56-0.88)
Dermatology	32.3	1.13 (0.81-1.58)	44.6	**0.68 **(0.50-0.93)	52.2	0.89 (0.65-1.21)	39.8	**0.67 **(0.49-0.92)	43.8	0.76 (0.56-1.04)
Rehabilitation	30.0	1.05 (0.73-1.50)	45.6	0.73 (0.52-1.01)	58.8	1.19 (0.85-1.66)	41.5	0.75 (0.53-1.05)	34.0	**0.51 **(0.36-0.72)
Gynaecology	30.5	1.02 (0.85-1.22)	50.9	0.86 (0.73-1.02)	28.0	**0.30 **(0.25-0.36)	45.4	**0.83 **(0.70-0.98)	46.6	0.85 (0.72-1.01)
Administration	26.2	0.94 (0.53-1.66)	56.9	1.27 (0.76-2.11)	46.2	0.76 (0.46-1.25)	41.5	0.84 (0.50-1.40)	38.5	0.66 (0.40-1.11)
Geriatrics	24.3	0.78 (0.49-1.24)	40.0	**0.58 **(0.39-0.88)	44.2	**0.66 **(0.44-0.99)	38.8	0.67 (0.44-1.02)	28.8	**0.40 **(0.26-0.62)
Infectious diseases	24.8	**0.67 **(0.51-0.89)	55.0	0.92 (0.71-1.18)	51.6	0.78 (0.61-1.00)	51.3	0.93 (0.72-1.19)	51.3	0.96 (0.75-1.23)
Ophthalmology	20.1	**0.60 **(0.45-0.81)	38.3	**0.53 **(0.42-0.68)	34.6	**0.43 **(0.34-0.56)	33.1	**0.51 **(0.39-0.66)	29.0	**0.40 **(0.31-0.52)
Child and adolescent	19.9	**0.59 **(0.44-0.79)	25.6	**0.29 **(0.22-0.37)	26.6	**0.29 **(0.22-0.38)	28.5	**0.40 **(0.31-0.52)	26.8	**0.36 **(0.28-0.47)
Surgery	21.8	**0.58 **(0.49-0.68)	44.4	**0.60 **(0.51-0.69)	41.5	**0.52 **(0.45-0.60)	40.0	**0.58 **(0.50-0.67)	40.8	**0.63 **(0.54-0.73)
Ear, nose, and throat	17.6	**0.50 **(0.38-0.66)	45.7	**0.72 **(0.58-0.90)	44.3	**0.65 **(0.52-0.81)	38.9	**0.65 **(0.52-0.82)	41.5	**0.71 **(0.57-0.89)
Oncology	14.7	**0.35 **(0.25-0.49)	38.2	**0.46 **(0.35-0.59)	50.6	**0.76 **(0.60-0.97)	31.2	**0.38 **(0.29-0.50)	27.0	**0.33 **(0.25-0.43)
Other	28.0	0.93 (0.76-1.13)	41.1	**0.58 **(0.48-0.70)	41.3	**0.57 **(0.47-0.69)	38.3	**0.62 **(0.51-0.75)	37.9	**0.61 **(0.51-0.74)

^a ^reference group (n = 1,874), ^b ^adjusted for rate of board certified specialists.

Bold figures indicates OR that is significantly greater or smaller than 1.

## Discussion

This is, so far, the internationally largest questionnaire study of physicians' sickness-certification tasks and problems, regarding type of situations they encountered and problems they experienced, including all physicians working in Sweden. Sickness-certification consultations were far more frequent than anticipated - more than two thirds of the physicians had such consultations, and of those over 80% had that at least once a week. Moreover, the rate of having such consultations was much higher in many other clinical settings than in PHC, e.g. in orthopaedics and in oncology. This has not been studied in other countries. Obviously, the previous focus on GPs regarding these tasks needs to be reconsidered. Nevertheless, a much higher rate of the physicians in PHC experienced these tasks as problematic. The majority of all physicians rated the task to assess magnitude of work incapacity and the prognosis of such incapacity as problematic. More detailed knowledge, regarding the broad tasks involved in sickness-certification consultations, has been called for [32,33]; some of that is provided here.

Strengths of the study are the very large sample size, that all physicians (N = 36,898) working and living in Sweden were included, that all clinical settings were included, and the detailed questions about these tasks. The study group is large enough to admit sub-group analysis, e.g. regarding different types of clinics. Another strength, from an intervention perspective, is that these results were based on the physicians' own experiences of tasks and problems. They can, therefore, be of good use when e.g. targeting different types of competence development [13]. A limitation is the drop out of 39%. Nevertheless, the response rate can be considered high for this type of study and the study design admits analyses of bias in the dropout. Differences in dropout rates between physicians being board certified specialist and non-specialist i.e. not yet fully trained or registered specialist might have affected our results. Non-specialists were, as expected, younger. During training, they often change residence, also geographically, which might be one reason for the higher drop out. The non-specialists had a higher dropout rate and also reported more problems regarding sickness certification than the specialists, which might have lowered the crude OR for problems reported from clinics with higher proportion of non-specialists. That is, in some cases the ORs might be underestimated, even when adjusted for rate of registered specialists.

To sickness certify a patient is a common task in health care in Sweden as in many other countries. However, this recommendation can so far not be based on scientific evidence [1]. In a previous, smaller study a slight association was found between physicians having at least six sickness-certification consultations a week and rates of having problems with this at least once a week [6]. The results of the present, much larger study, goes in the same direction and we found similar associations in the majority of clinics, however, not for physicians in oncology and PHC. The proportion of physicians experiencing problems regarding sickness-certification in general, as for specific items, varied with type of clinic. The physicians in PHC had the highest ORs for experiencing problems, although they did not have the highest frequency of sickness-certification consultations.

Actually, also some physicians in geriatrics and child care had sickness-certification cases. This can be explained in at least three ways; one is that the participants were asked to indicate the type of clinic where they *mainly *worked, but some might also be clinically active in other types of clinics. The second is that some of their patients are adolescents, above the age of 16, and thus can be sickness absent and in geriatrics also some patients work in spite of old age. The third is that they might sickness certify parents of children or relatives of geriatric patients. So far, none have studied this.

We were surprised by the very high rates of consultations and also by the large variety in rates of physicians experiencing problems with these tasks. In Sweden, as in most welfare countries, specialists generally are to refer patients to PHC when treatment is finished or stabilised. It is an understanding that sickness-certification is to be monitored from PHC if a patient is referred to other clinics by the general practitioner in PHC. Nevertheless, very high rates of physicians in different clinics had such tasks very often. More studies are needed to verify these results also in other countries. However, other studies indicate that GPs generate about half of all sickness-certification, which is well in line with our results [1,34]. An obvious issue here is whether the results can be generalized also to other countries. Sickness-certification practices have only been studied in very limited populations and mainly for GPs in other countries - thus, the situation might be similar there, however, that remains to be seen. Regarding other aspects of sickness-certification practice, results from different countries and from different time periods have been unexpectedly similar [11].

The most problematic part of sickness-certification seemed to be to assess the magnitude of the patient's work capacity. This result goes in line with some previous studies [19,35,36]. A variety of instruments for assessment of work capacity are used in different countries, however, scientific knowledge on the validity of them, their effects, and on possible implications for the work of physicians is warranted [37]. Overall, the highest OR for problems with work-capacity assessments was found among physicians in PHC and in psychiatry, rheumatology, and neurology which had a still higher OR for problems providing a long-term prognosis of work capacity. We have not found any other studies about this.

The majority of physicians had sickness-certification consultations every week and problems experienced regarding this varied substantially in frequency as well as severity between clinical settings. The physicians at a vast majority of the clinics regarded sickness-certification consultations as problematic and far more so among physicians in PHC in spite of that they did not have as many such consultations. So far, most interventions concerning sickness-certification have been targeted towards physicians in PHC/GPs. Other physician groups with high frequencies of consultations and/or problems were found in oncology, orthopaedic, psychiatric, pain management, and rheumatologic clinics. The results indicate the importance to take account of the variety of problems in physicians in different clinical settings experience, when planning interventions aimed at improving their work with sickness-certification of patients.

## Conclusions

So far, most interventions regarding physicians' sickness-certification practices have been targeted towards PHC and GPs. Our results indicate that those physicians also to a great extent experience sickness-certification consultations as problematic. Nevertheless, also other physicians have high risks for experiencing them as problematic, e.g. in rheumatology, neurology, psychiatry, and orthopaedic clinics. Moreover, in several clinical settings the physicians had such consultations more often than the GPs. Thus, the results indicate that much can be gained through focusing on physicians also in other types of clinics when planning interventions to improve physicians' sickness-certification practice.

## Competing interests

The authors declare that they have no competing interests.

## Authors' contributions

The authors of this manuscript are members of a research project investigating physicians' sickness-certification practice. CL contributed in the study design, data collection, interpretation, drafting, and writing the manuscript, and supervision. BA and GN contributed to the conception, design of the study, interpretation of the data, and revisions of the manuscript. EH performed the statistical analyses and contributed to interpretation and revisions of the manuscript. AE and AL contributed to the data analysis and interpretation as well as revisions of the manuscript. YS contributed to interpretation and revisions of the manuscript. KA contributed to the conception, design of the study, data collection, interpretation of the data, revisions of the manuscript and supervision. All authors have read and approved the final manuscript.

## Pre-publication history

The pre-publication history for this paper can be accessed here:

http://www.biomedcentral.com/1471-2458/10/752/prepub
